# Evaluating the Effects of LDL Cholesterol Levels on Tau Visualization in [F‐18]flortaucipir PET Scans

**DOI:** 10.1002/alz.090019

**Published:** 2025-01-09

**Authors:** Jaipal Virdi, Daniel H. Silverman

**Affiliations:** ^1^ University of California, Los Angeles, Los Angeles, CA USA

## Abstract

**Background:**

Alzheimer's Disease and other neurodegenerative diseases are characterized by abnormal tau protein accumulation in the brain. PET imaging utilizing the [F‐18]flortaucipir tracer is a widely used method for visualizing such conditions, yet its effectiveness can be compromised by off‐target binding. To shed light on this issue, our study focuses on how elevated cholesterol concentrations of low‐density lipoproteins (LDL) and standard uptake values (SUVR) from corresponding tau‐PET scans may influence the efficacy of [F‐18]flortaucipir.

**Method:**

Demographic, metabolic, and imaging data were sourced from the Alzheimer's Disease Neuroimaging Initiative for 56 subjects at varying cognitive statuses (CN, n=24; SMC, n=4; MCI, n=25, and AD, n=3). This included t1‐weighted MRI scans and tau‐PET scans acquired with [F‐18]flortauicipir. Cholesterol levels were calculated using the ratio of cholesterol to total lipids in the LDL. Image processing was conducted using Clinica software, with volumes of interest mapped using SUVR from the Hammers atlas.

**Result:**

Our analysis revealed significant correlations between LDL cholesterol levels and radiotracer uptake in the right & left caudate and accumbens nuclei (r=0.45, p=0.00046; r=0.45, p=0.00049; r=0.28, p=0.032, and r=0.26 p=0.05 respectively). When subjects were stratified with respect to the presence (n=27) or absence (n=29) of the APOE4 allele, some interaction was observed for the right caudate nucleus, which became somewhat more tightly correlated to the LDL parameter among those with the ε4 genotype (r=0.53, p=0.003).

**Conclusion:**

A plausible mechanism explaining the correlations found and [F‐18]flortaucipir off‐targeting binding as a whole may be that oxidized low‐density lipoproteins (oxLDL), which proliferate as concentrations rise, could prompt microglia to transform into lipid‐laden foam cells. These cells could then create hydrophobic pockets that slow the radiotracer washout, with this effect being localized to the basal ganglia. This aligns with other research that has identified heightened oxLDL levels to specifically affect the perivascular spaces in the basal ganglia. Furthermore, our findings are consistent with previous studies that have shown increased microglial inflammation associated with the APOE4 genotype.

